# Natural antibodies and CRP drive anaphylatoxin production by urate crystals

**DOI:** 10.1038/s41598-022-08311-z

**Published:** 2022-03-16

**Authors:** Anne Kathrin Wessig, Leonie Hoffmeister, Annika Klingberg, Anika Alberts, Andreas Pich, Korbinian Brand, Torsten Witte, Konstantin Neumann

**Affiliations:** 1 Institute of Clinical Chemistry, Hannover Medical School, 30625 Hannover, Germany; 2 Research Core Unit Proteomics & Institute of Toxicology, Hannover Medical School, 30625 Hannover, Germany; 3 Department of Rheumatology and Immunology, Hannover Medical School, 30625 Hannover, Germany

**Keywords:** Acute inflammation, Complement cascade, Innate immunity, Crystal deposition arthropathies

## Abstract

In gout, crystallization of uric acid in the form of monosodium urate (MSU) leads to a painful inflammatory response. MSU crystals induce inflammation by activating the complement system and various immune cell types, and by inducing necrotic cell death. We previously found that the soluble pattern recognition molecule C-reactive protein (CRP) recognizes MSU crystals, while enhancing complement activation. In the absence of CRP, MSU crystals still induced complement activation, suggesting additional CRP-independent mechanisms of complement activation. In the present study, we searched for additional MSU crystal-binding complement activators. We found that all healthy individuals, even unborn children, have MSU crystal-specific immunoglobulin M (IgM) in their blood. This indicates that innate IgM, also known as natural IgM, recognizes these crystals. In serum lacking IgM and CRP, MSU crystals showed negligible complement activation as assessed by the production of the anaphylatoxins C4a, C3a, and C5a (listed in order of production via the classical complement pathway). We show that IgM and CRP both activate the classical complement pathway on MSU crystals. CRP was more efficient at fixating active C1 on the crystals and inducing release of the most inflammatory anaphylatoxin C5a, indicating non-redundant functions of CRP. Notably, while CRP recognizes MSU crystals but not the related calcium pyrophosphate dihydrate (CPPD) crystals, natural IgM bound to both, suggesting common and distinct mechanisms of recognition of individual crystal types by complement activators.

## Introduction

Deposition of crystals within joints can lead to the development of arthritis. While crystallization of uric acid in the form of monosodium urate (MSU) leads to gout, the symptomatically similar disease, pseudogout, is caused by the formation of calcium pyrophosphate dihydrate (CPPD) crystals. Both crystal types are inducing sterile inflammation by directly activating immune cells^1–4^. Crystals also indirectly stimulate and recruit immune cells by activating the complement system^5,6^ and by inducing cell death, which leads to release of danger-associated molecular patterns that further drive the inflammatory response^7,8^. Together these mechanisms cause a sterile inflammatory response that is hardly distinguishable from a microbe induced inflammation. Bacterial infection is thus the most important differential diagnosis of acute gout^3^.

The complement system is part of the humoral immune system^9^. It is characterized by the sequential activation (proteolytic cleavage) of its protein components upon binding to a surface, e.g., of bacteria. Three main pathways for complement activation have been described, which converge at the activation of the central complement component C3: (1) the classical pathway relies mainly on binding of complement activators like IgM, IgG, or CRP, which recruit and activate complement factor C1. (2) The lectin pathway is C1-independently initiating the cascade through MBL, ficolins, or IgA, activating MASP-1/2. Both classical and lectin pathway activate C2 and C4 to generate a C3 convertase. (3) The alternative pathway relies on auto-activation of complement protein C3 which is enhanced by other factors (e.g., properdin)^10^. Activated C3 then leads to cleavage of C5 to induce formation of the membrane attack complex consisting of factors C5b-9 (also called terminal complement complex), which forms pores in the membrane of attacked cells. Activation (by proteolytic cleavage) of C4, C3, and C5 leads to release of inflammatory peptides C4a, C3a, and C5a, which are collectively called anaphylatoxins. They activate distinct receptors and lead to distinct responses. While C4a mainly enhances endothelial permeability^11^, C3a and C5a act chemotactic for immune cells, activate mast cells, or induce inflammatory gene expression. The membrane attack complex can also be released in its soluble form (sC5b-9) and induce immune cell activation^12^.

As early as 1973, Naff and Byers found that MSU crystals activate the complement system^13,14^. In normal human serum, a functional C5 convertase assembles on the surface of MSU crystals and cleaves C5 to generate anaphylatoxin C5a and sC5b-9^5^. C5a generated by MSU crystals was found to enhance IL-1β production in vitro^15^. Recently, mice deficient for the central complement component C3 or the receptor for anaphylatoxin C5a were shown to exhibit strongly reduced neutrophil influx and IL-1β production upon intraperitoneal injection of MSU crystals^16,17^. C6-deficient rabbits showed reduced MSU crystal-induced inflammation suggesting C5b-9 may also be involved^18^. MSU crystals activate the classical complement pathway independently of IgG, which was believed to depend on binding of C1 directly to the crystals^19^, while one report showed that complement activation (C3 cleavage) could be enhanced by addition of CRP and IgG^20^. Others found that the alternative pathway contributes to MSU-dependent complement activation^21,22^. CPPD crystals also activate the alternative complement pathway. Complement activators recognizing CPPD crystals are unknown.

To recognize microbes, the complement system uses innate complement activators that recognize conserved microbial patterns, called pattern recognition molecules, or antibodies that have been raised by the adaptive immune system against a specific microbe or microbial structure^9,23^. It is unclear if the complement system uses similar mechanisms to recognize crystals.

We recently purified MSU crystal-binding proteins and found that C-reactive protein (CRP) is one of the strongest MSU crystal-binding proteins in human body fluids^24^. As an acute phase protein, CRP concentration in the blood strongly increases during inflammation, e.g., during gout flares. We showed that CRP is required for fixation of active C1 of the classical and active MASP-1 of the lectin complement pathway on the surface of MSU crystals. We also showed enhanced fixation of C3 and C5b-9 in the presence of CRP suggesting CRP principally activates the complete complement pathway on the surface of MSU crystals. Similarly, CRP had been found before to bind to cholesterol crystals to activate the complement system^25^, which suggests that CRP may be a more general crystal-recognition molecule. In the absence of CRP, complement activation by both MSU and cholesterol crystals was partly preserved, so CRP-independent mechanisms of complement activation exist. CPPD crystals are special in that they are not recognized by CRP^24^, while still activating the complement system.

In this study, we set out to find the additional complement activators for MSU crystals. We found a second innate immune sensor (natural IgM) that binds to both MSU and CPPD crystals to drive CRP-independent complement activation, and we compared the ability of IgM and CRP to induce production of the anaphylatoxins C4a, C3a, and C5a.

## Results

### MSU crystal-specific antibodies in healthy individuals

**Figure 1:**
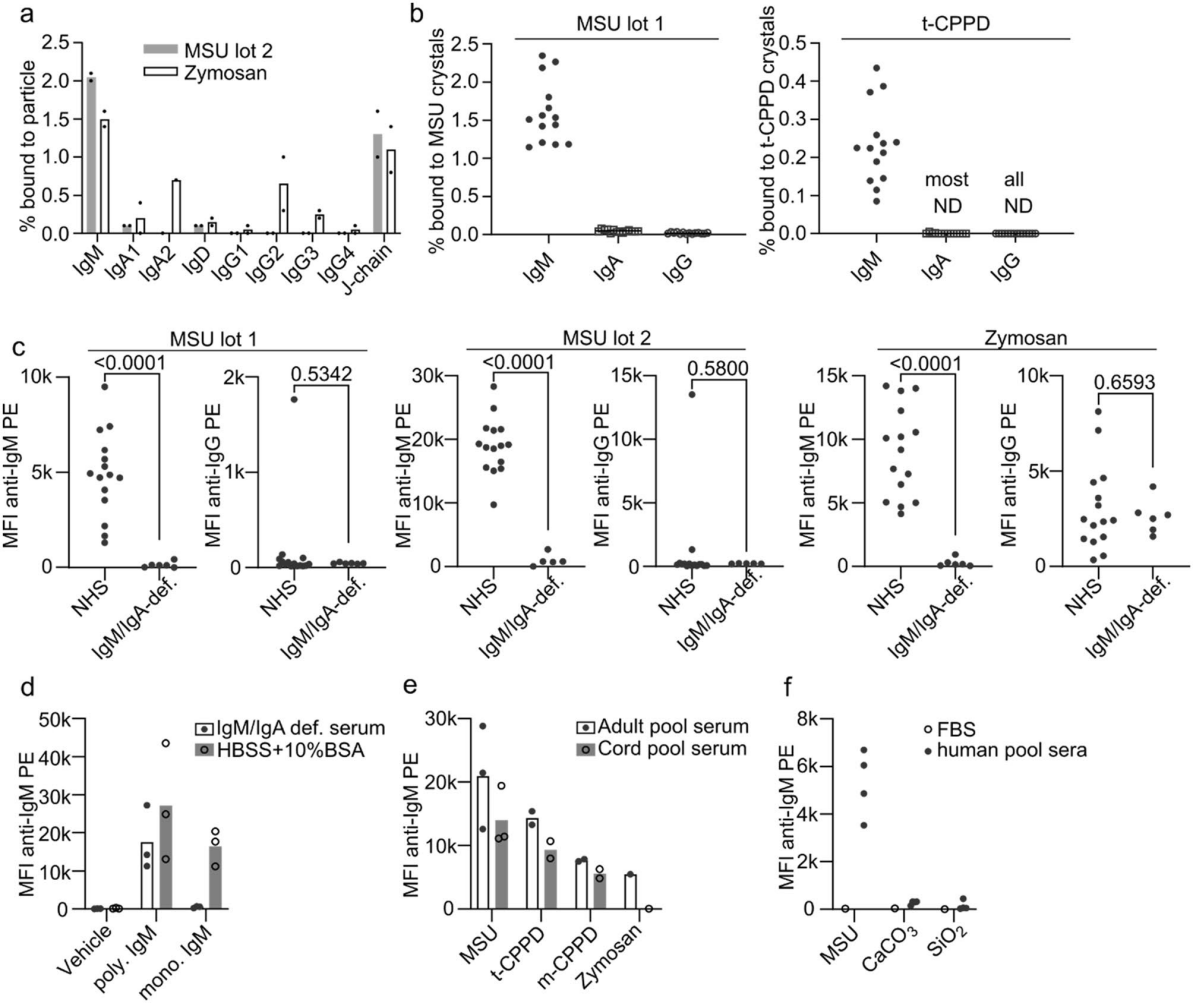
Natural IgM binds to MSU and CPPD crystals. (**a**) Serum proteins bound to each indicated particle were analyzed by LC–MS. Fraction of each antibody isotype bound to each indicated particle is shown. Data from 2 individual serum samples is depicted. In one serum sample, IgA2 was not detected; therefore, only one data point is shown. (**b**) Fraction of immunoglobulin isotypes bound to MSU or t-CPPD crystals from 14 normal human serum samples quantified from Western blot analysis. ND = not detected. Raw data (blot images) is shown in Supplementary Fig. 1. (**c**) Individual normal human sera (NHS, including samples from B) or IgM/IgA-deficient serum samples were incubated with the indicated particles, and bound IgM and IgG were detected using anti-IgM PE or anti-IgG PE, respectively. Median fluorescence intensity (MFI) of the particles was normalized by subtracting the MFI of the negative control (FBS). Means of the NHS and IgM/IgA-deficient serum samples were compared by an unpaired t-test. (**d**) Binding of purified polyclonal (poly.) or monoclonal (mono.) IgM to three distinct preparations of MSU crystals in IgM/IgA-def. serum or HBSS + 10% BSA. (**e**) Pooled human serum collected either from healthy adults or from human cord blood was incubated with the indicated particles, and bound IgM was detected as in (c). Values for each particle preparation and the mean are shown. (**f**) MSU (lot 2), silica (SiO_2_), or calcium carbonate (CaCO_3_) crystals were incubated in FBS or 4 normal human pool serum samples. Bound human IgM was detected as in (c) and MFI for each serum sample is shown including the negative controls (FBS).

As described above, we had previously identified MSU crystal-binding proteins by liquid chromatography–coupled mass spectrometry (LC–MS). As a control particle we had used zymosan, a fungal cell wall preparation from *S. cerevisiae* containing mainly β-glucans. We had incubated both MSU crystals and zymosan in human serum, washed away the unbound proteins, eluted the bound proteins, and subjected them to LC-MS^26^. Among the bound proteins, we had found CRP and the related pentraxin serum amyloid P (SAP)^24^. So far, we were unable to show a role for SAP in complement activation by MSU crystals (data not shown). Thus, we searched for other complement activating proteins within the identified crystal-binding proteins. As antibodies are common activators of complement, we analyzed the fraction of each immunoglobulin isotype bound to MSU crystals and zymosan (Fig. 1a, “Supplementary Dataset”). All isotypes showed some binding to zymosan, likely due to previous immune responses to ubiquitous fungi. IgM was the only isotype showing strong binding to both zymosan and MSU crystals. Consequently, the J-chain, which links IgM and IgA monomers, was also found on both MSU crystals and zymosan. Since these data were based only on two donors, we next purified proteins that bound to MSU from 14 human serum samples obtained from healthy individuals (aged 20–65 years). We performed Western blot analysis to determine the relative amount of bound IgM, IgA, and IgG. Densitometric quantification of the blots showed that, compared to IgA and IgG, a much higher fraction of IgM bound to MSU crystals (Fig. 1b, Supplementary Fig. 1). We tested the same for the related CPPD crystals (here, triclinic (t) crystals were used), which showed similar results, albeit the fraction of bound IgM was lower. Binding of IgM to both crystal types was found in every serum sample tested with low variability, indicating that most, if not all individuals have naturally occurring MSU- and t-CPPD-specific IgM antibodies. We confirmed this observation using a flow cytometer. Two independent preparations of MSU crystals preincubated in normal human sera showed a strong signal after staining with a fluorescent anti-IgM antibody, which was comparable to zymosan particles (Fig. 1c). As a control we used serum samples obtained from patients with common variable immunodeficiency (CVID), which were selected based on very low IgM concentrations (< 0.05 mg/ml; normal range 0.4–2.3 mg/ml^27^). IgA was also low in these IgM-deficient sera, while IgG levels were in the normal range. In these IgM/IgA-deficient serum samples very low signals were obtained, showing the specificity of the assay. Staining with anti-IgG showed again more IgG binding to zymosan than to MSU crystals. However, in this analysis using a flow cytometer, one of the normal human serum samples also showed binding of IgG to both MSU crystal preparations, suggesting that weaker interactions are retained in this assay. Next, we tested if IgM binds directly to the crystals or indirectly via other serum proteins. IgM purified from healthy individuals (polyclonal IgM) showed slightly stronger binding in serum-free albumin (BSA) solution than in IgM-deficient serum, indicating that in principle IgM can bind directly to the crystals (Fig. 1d). A monoclonal IgM (purified from a myeloma patient) did not bind to the crystals in IgM-deficient serum, suggesting that the specificity of the IgM antigen binding site is relevant. It, however, also showed binding in BSA solution, which is likely due to the absence of any competing crystal-binding proteins like ApoB^24,26,28^. If IgM binds exclusively directly to the crystals, blocking the surface by preincubating the crystals with IgM-deficient serum should reduce binding of IgM. After preincubation of MSU crystals with IgM-deficient serum, binding of IgM from human normal pool serum was, however, not consistently reduced (Supplementary Fig. 2b). Thus, IgM may not only bind directly but also indirectly to the crystals. Finally, to test whether crystal-specific IgM was natural, i.e., it is innate and not produced after encountering a foreign antigen, we compared IgM binding to crystals in pooled serum collected either from adults or from cord blood samples. The binding of IgM to three independent preparations of MSU and two independent preparations of both t-CPPD and monoclinic (m)-CPPD in cord blood serum was nearly as strong as that in adult serum (Fig. 1e). No zymosan-specific IgM was detectable in cord blood serum. Thus, natural IgM recognizes MSU and CPPD crystals, while zymosan-specific IgM is likely generated in response to foreign antigens. Together, these findings show that most, if not all individuals have MSU-specific IgM in their blood, which is at least partly innate. To test, if IgM binds to all types of crystals or particles, we analyzed binding of IgM in human pool serum to calcium carbonate (CaCO_3_), silica particles (SiO_2_), and cholesterol crystals. IgM only weakly bound to these particles as compared to MSU crystals (Fig. 1f, Supplementary Fig. 2c), suggesting IgM only recognizes a subset of crystals (as does CRP).

### IgM-dependent complement activation by MSU crystals

To test the impact of IgM on crystal-induced complement activation, we used the same serum samples obtained from CVID patients with serum IgM concentrations below 0.05 mg/ml.

**Figure 2:**
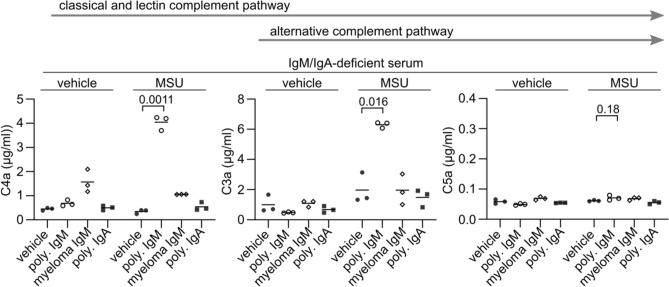
Polyclonal IgM activates complement in the presence of MSU crystals. IgM/IgA-deficient serum obtained from three individuals was depleted of CRP and then reconstituted with polyclonal IgM, IgA, or monoclonal IgM at 0.4 mg/ml. Serum samples were incubated for 30 min at 37 °C with vehicle or MSU (lot 1, 20 mg/ml). The concentrations of anaphylatoxins C4a, C3a, and C5a are shown. Lines represent the means. p values for comparisons of the means were calculated using paired t-test. Arrows above the graphs indicate the sequence of production of C4, C3a, and C5a in the three main complement pathways.

We reconstituted IgM/IgA-deficient serum with polyclonal and monoclonal IgM that we already used in Fig. 1d and analyzed MSU crystal-induced complement activation by measuring the complement activation products C4a, C3a, and C5a. Only polyclonal IgM induced C4a and C3a production in the presence of MSU crystals (Fig. 2), which is in line with above findings that only polyclonal IgM bound to the crystals in serum (Fig. 1d). However, at the concentrations used, IgM was not able to induce significant amounts of C5a, while polyclonal IgA did not induce any anaphylatoxin production.

### CRP-mediated anaphylatoxin production in the presence and absence of IgM

We started this study to find the complement activator that can compensate for CRP in its absence. To test if CRP has non-redundant roles in complement activation in the presence or absence of the identified second complement activator IgM, we reconstituted normal human pool sera or IgM/IgA-deficient sera with CRP. We analyzed the production of anaphylatoxins (C4a, C3a, and C5a) and the final product of the pathway (sC5b-9) after incubation with MSU or t-CPPD crystals, which are not recognized by CRP, as a control.

**Figure 3:**
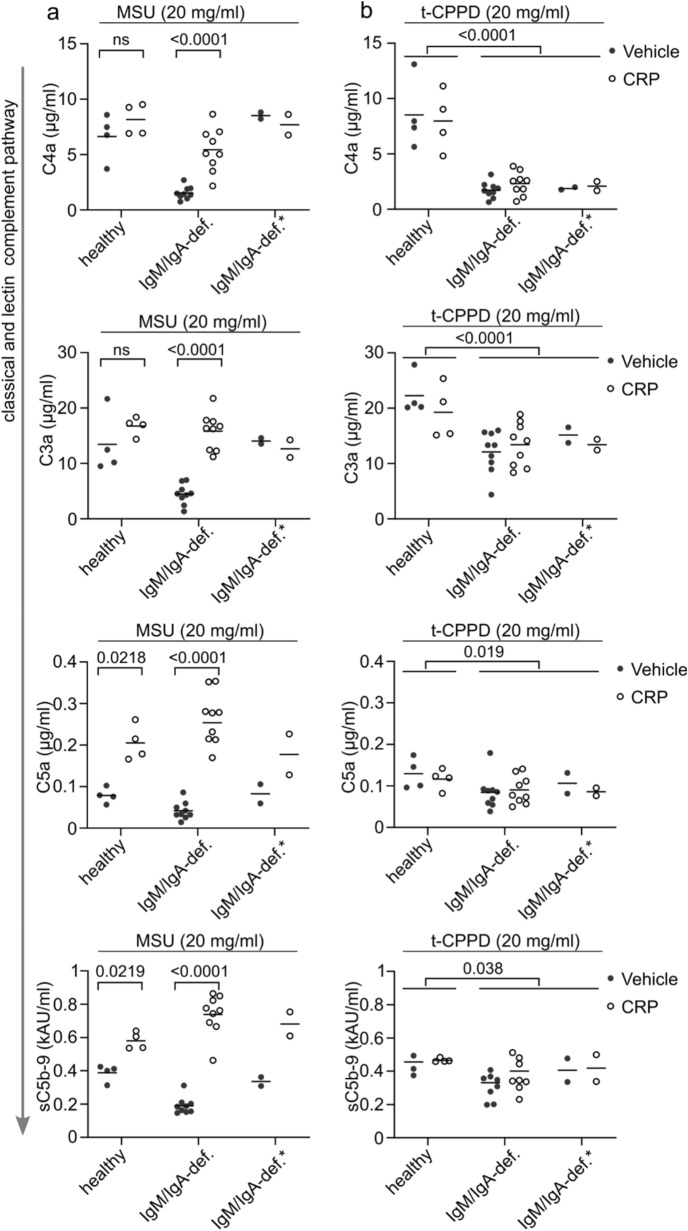
Non-redundant role of CRP in complement activation by MSU crystals. Four pooled serum samples obtained from healthy individuals and 11 IgM/IgA-deficient serum samples obtained from CVID patients were depleted of residual CRP and reconstituted with CRP (30 µg/ml) or vehicle. Sera were incubated with either MSU lot 1 (**a**) or t-CPPD (**b**) for 30 min at 37 °C. Concentrations of soluble complement system activation products C4a, C3a, C5a, and sC5b-9 in the supernatant are shown. Two serum samples from CVID patients that showed early classical pathway activation are displayed separately (IgM/IgA-def.*). Lines represent the means. For comparison of serum samples with or without CRP, p values were calculated by paired t-test; for comparison of IgM/IgA-sufficient and IgM/IgA-deficient serum samples, an unpaired t-test was used.

In pool sera from healthy individuals, CRP did not significantly enhance C4a and C3a production (the earliest products of the classical complement pathway) but enhanced the production of C5a and sC5b-9 (Fig. 3a). In the absence of IgM, IgA, and CRP, all complement activation products were hardly present, but their production could be fully reconstituted by the addition of CRP, indicating IgM is indeed the second independent complement activator that we were looking for. The production of the later activation products C5a and sC5b-9 was reconstituted even above values achieved in normal human serum, indicating CRP is especially efficient at inducing later complement activation events. Two of the 11 IgM/IgA-deficient serum samples, however, behaved like normal IgM/IgA-sufficient serum samples (with CRP-independent C4a and C3a generation) and are denoted as “IgM/IgA-def.*” in Fig. 3. These sera had no detectable level of CRP and only very low level of MSU crystal-binding IgM (Supplementary Fig. 3), so either a third IgM- and CRP-independent pathway exists or residual antibodies in the two serum samples have been sufficient to activate the early components of the complement pathways.

To show the specificity of CRP-induced complement activation, we also tested complement activation by t-CPPD crystals (which are not recognized by CRP^24^) (Fig. 3b). Both in normal pool sera and in IgM/IgA-deficient sera, CRP did not alter production of any of the complement activation products (Fig. 3b), confirming the specificity of CRP for MSU crystals. However, a significant reduction in C4a and to a lower extend in C3a levels in the IgM/IgA-deficient serum samples was observed, indicating that IgM also activates the classical complement pathway on t-CPPD crystals, while additional IgM- and CRP-independent mechanisms for complement activation by t-CPPD seem to exist.

**Figure 4:**
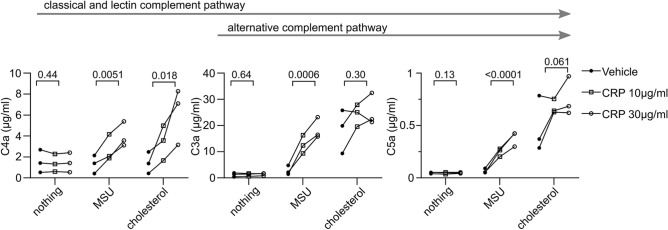
CRP drives anaphylatoxin production in the presence of MSU crystals. IgM/IgA-deficient serum obtained from 3 individuals with CVID was depleted of CRP and then reconstituted with vehicle or CRP at 10 or 30 µg/ml. The serum samples were incubated for 30 min with nothing, MSU lot 2, or cholesterol crystals. The concentrations of anaphylatoxins C4a, C3a, and C5a in the serum after the incubation are shown. p values are shown for the comparison of the means of vehicle, 10 µg/ml CRP, and 30 µg/ml CRP as determined by one-way repeated-measures ANOVA.

To verify that CRP-mediated complement activation is dependent on the presence of MSU crystals, we repeated the experiment with three IgM/IgA-deficient sera and incubated them without crystals, MSU crystals, and cholesterol crystals, which are also recognized by CRP (Fig. 4). CRP did not induce anaphylatoxin production in the absence of crystals, and MSU crystal-induced anaphylatoxin production was largely dependent on CRP in these sera. In line with previous reports^25^, CRP induced C4a generation by cholesterol crystals, while some C3a and C5a production was seen in the absence of CRP, likely activated by redundant complement activators^6,29,30^.

### Activation of C1s by IgM and CRP

**Figure 5:**
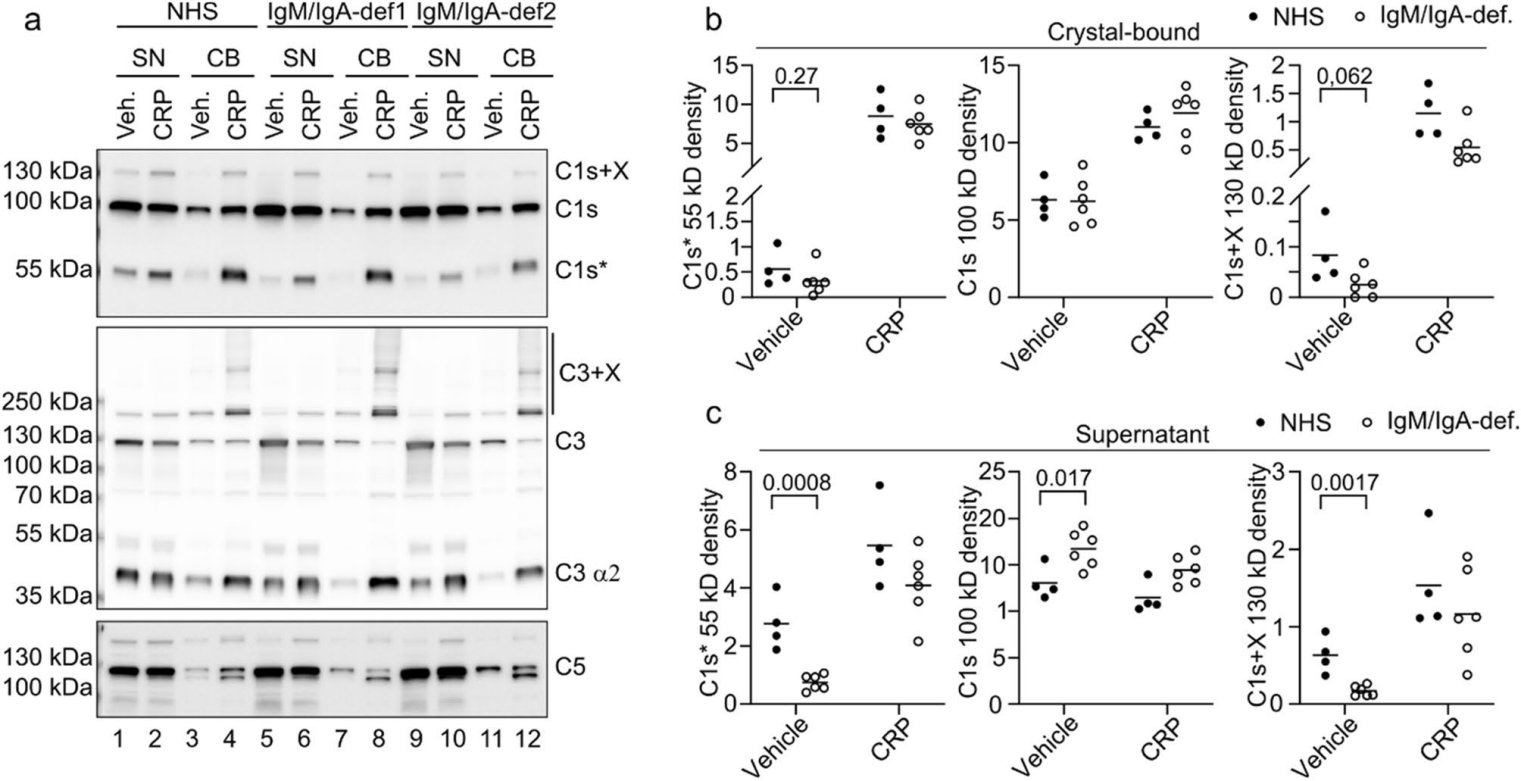
Activation of C1s by IgM and CRP. (**a**) Representative Western blot of supernatants (SN) or eluted crystal-bound (CB) proteins using the indicated antibodies (activated cleavage products are marked with an asterisk*, + X denotes bands that have an apparent molecular weight higher than the full-length protein). Uncropped images and images without enhanced contrast are supplied in the Supplementary Figs. 4 and 5. (**b**, **c**) Densitometric analysis of Western blots of C1s bands within crystal-bound proteins (**b**) and within the supernatants (**c**) of 4 normal pool sera and 6 IgM/IgA-deficient serum samples incubated with MSU crystals (samples from Fig. 3).

In our previous study, we showed that CRP was required for fixation of active complement factors C1s (the catalytic subunit of C1) on the surface of MSU crystals even in IgM-sufficient serum^24^. Since IgM is known to activate the classical pathway via C1, we analyzed C1s, C3, and C5 fixation on MSU crystals in the absence of IgM (samples from Fig. 3a). One representative Western blot image of a normal serum and two IgM/IgA-deficient sera is shown in Fig. 5a (the remaining Western blots used for quantification are shown in Supplementary Fig. 4). In line with our previous data^24^ and the ability of CRP to induce the release of C5a, the addition of CRP induced fixation of cleaved versions of C1s, C3, and C5 on MSU crystals (in both normal and IgM/IgA-deficient sera). C1s is activated by cleavage and one of the cleavage products is observed at 55 kDa (denoted as C1s*). Upon activation, we always observed another band of C1s, which has an apparent molecular weight higher than full length C1s (denoted C1s + X). As it is stable in reducing SDS-PAGE, it is likely a fusion with another protein via a peptide bond. In the absence of IgM/IgA, we did not see reduced binding of the activated C1s. When we quantified the intensity of the band of activated C1s* observed on blots of four normal and six IgM/IgA-deficient serum samples, the difference was not statistically significant (Fig. 5b, left panel). We also quantified the C1s + X activation product: no statistically significant difference between IgM-sufficient and IgM-deficient serum was found (Fig. 5b, right panel). However, when we evaluated active C1s* released into the supernatant and not fixated on crystals, we found strongly increased activated C1s* in the presence of IgM (Fig. 5c, left panel). Similarly, the larger activation product (C1s + X) was strongly increased in presence of IgM (Fig. 5c, right panel). This was not due to different levels of C1s in the sera, as the normal full length C1s was even reduced in the presence of IgM (Fig. 5c, middle panel). Similarly, when we added polyclonal IgM to two IgM/IgA-deficient sera, activated C1s was again detectable after incubation with MSU crystals (Supplementary Fig. 6). Together, this suggests that IgM activates C1s on the surface of MSU crystals but cannot fixate it as efficiently as CRP. Accordingly, fixation of C3 and C5 was largely independent of IgM (Fig. 5a, Supplementary Fig. 5).

In summary, we have identified natural IgM as a CRP-independent complement activator for MSU crystals, while we show that CRP is more efficient at fixating complement factors on the crystals and inducing later complement activation products C5a and sC5b-9.

## Discussion

Inflammation during a gout flare is hardly distinguishable from inflammation induced by microbes. Thus, it is likely driven by the same pattern recognition molecules/receptors as microbe-induced inflammation. What remains unclear so far is if these receptors specifically recognize the crystals. The NLRP3 inflammasome is a main driver of crystal-induced inflammation, but it does not seem to interact with crystals but rather senses damage induced by the crystals to plasma membrane or lysosome^31,32^. Uptake of crystals not necessarily requires specific receptors either, as the crystals directly interact with the plasma membrane^33,34^. However, several transmembrane receptors have been shown to recognize MSU crystals rather specifically and/or to be involved in MSU crystal-induced inflammation^26,35–39^. Additionally, we found a remarkably specific binding of CRP to MSU crystals but not the related CPPD crystals, suggesting soluble pattern recognition molecules recognize crystal surfaces. In this study, we identify natural IgM as a second soluble molecule for MSU crystal recognition by the complement system. Most proteins that show strong binding to MSU crystals have in common that they have multiple binding sites: CRP and IgM are homopentamers, MARCO is a homotrimer, and Clec12A oligomerizes^40^. Multiple interactions of low affinity may lead to a high avidity interaction of the whole molecule with the crystal surface, which is probably necessary since a classical key-lock recognition mechanism seems unlikely for the various crystal surfaces. In line with this notion, the CRP related pentraxin SAP and ficolin 2, both pentamers bind to cholesterol^25,30^, and we found both also on the surface of MSU crystals^24^.

Natural IgM is believed to be produced by B1 B-cells independent of any foreign antigens, presumably in response to self-antigens^41^. It has been shown not only to bind various microbes but also to play a role in complement activation by necrotic cells and the heparin:PF4 complex, which is targeted in heparin-induced thrombocytopenia^42–44^. Other “naturally occurring” antibodies arise from the poly-reactivity or cross-reactivity of IgM produced in infancy in response to ubiquitous microbes, as has been established for antibodies against ABO blood type antigens^45^. We found similar binding of IgM to MSU crystals incubated in cord blood or adult serum, suggesting that the MSU-specific IgM in adult serum is natural. As we cannot differentiate between the origins of the MSU crystal-binding IgM in adult serum, we also cannot exclude the possibility that IgM raised against foreign antigens contributes to the pool of crystal-binding IgM in adult serum. Still, natural IgM recognizes MSU crystals and unlike CRP also binds to CPPD crystals.

As expected, we found that both IgM and CRP activate the classical complement pathway on the surface of MSU crystals. Notably, IgM was less efficient at fixating complement factors on the surface of the crystals. This could be due to a weaker or unstable binding of IgM to the crystals or partly indirect binding. While we showed that IgM is in principle capable of binding the crystals directly (Fig. 1d), it may still bind indirectly in the presence of crystal-coating serum components. We tried to block IgM-binding by coating the crystals with serum or purified proteins, but the results were variable and donor specific. Further research is thus needed to clarify the binding mechanism of IgM to crystals. IgM has been shown before to weakly bind to cholesterol crystals in normal human serum. Intriguingly, IgM bound more strongly in the absence of C1q^30^. It will be interesting to see, if absence of C1q also enhances binding of IgM to MSU and CPPD crystals.

CRP binds directly to the crystals and is especially efficient at inducing the late complement activation products C5a and sC5b-9. Both C5a and C5b-9 have been shown to be involved in MSU-induced inflammation in animal models. C5a acts as a chemoattractant and can induce expression of inflammatory genes like proIL-1β^16,17^, and C5b-9 may activate the NLRP3 inflammasome in bystander cells^18,46–48^. Thus, it is likely that high CRP levels during an acute phase reaction increase the likelihood of the initiation of a gout attack.

Before our findings, CRP had already been shown to recognize cholesterol crystals to activate the complement system, and to co-localize with C5b-9 complexes in human atherosclerotic plaques^25^. Thus, also on those crystals, CRP drives the complement system to the very end. This is in contrast to other ligands of CRP, where CRP additionally recruits the inhibitory complement factor H (CFH) to stop complement activation beyond C3^49^. This does not appear to happen on MSU and cholesterol crystals for yet unknown reasons.

Cholesterol crystals additionally activate the alternative pathway^6,29^, the lectin pathway via ficolin-2, and recruit C1q via IgM^30^, showing some overlap with MSU crystals. Two early reports also demonstrated alternative pathway activation by MSU crystals^21,22^. Whether this requires CRP or IgM remains to be seen. Recently, it was shown that several nanoparticles activate the alternative pathway via IgG^50^. This may also happen on MSU crystals, depending on the presence of MSU crystal-binding IgG. We have found very low levels of MSU crystal-binding IgG in most individuals, at least when tested in serum. In line with earlier results^51^, we found that purified IgG binds to MSU crystals in the absence of competing serum proteins (data not shown), similar to the unspecific monoclonal IgM we used in this study (Fig. 1d). This may become relevant in body fluids low in serum protein (mainly apolipoproteins^26,52,53^), or under certain experimental conditions. One report showed that addition of IgG could enhance complement activation by MSU crystals^20^. Thus, it remains to be seen under which conditions IgG activates complement on MSU crystals. It is possible that gout patients develop MSU crystal-specific IgG as MSU crystals may act as antigens for an immune response, or that there are individuals with autoantibodies reacting with serum proteins that coat the crystals. At least one serum we used in our study showed IgG binding to MSU crystals. So, it may be worthwhile to screen patients for these antibodies to find out if these antibodies are associated with a medical condition and what they bind to: crystals or coated proteins.

In two out of eleven IgM/IgA-deficient sera in which CRP was depleted, early complement activation was preserved (Fig. 3a). These may have contained residual IgM, sufficient crystal-binding IgG, or a completely independent complement activator. Thus, there could be a third complement activator for MSU crystals, which we may search for in a future study.

Besides activation of the complement system, binding of CRP or IgM to crystals may alter gout pathophysiology also by other mechanisms. In mice, MSU crystal-binding IgM are required for precipitation of uric acid and its adjuvant properties^54^, while the pentraxin PTX3 has been shown to inhibit crystallization of calcium oxalate^55^. It remains to be seen if CRP or human IgM also alter crystallization of urates, the prerequisite for the development of gout.

## Methods

### Human serum and buffers

We used left-over diagnostic samples of serum obtained from healthy individuals (age range 20–65 years, 9 female and 5 male) and 11 CVID patients who showed IgM and IgA levels below 0.05 g/l (age range 31–55 years, 5 female and 6 male). All serum samples had IgG levels in the reference range, while we did not inquire in which cases this was due to immunoglobulin replacement therapy. Most sera were collected before any SARS-CoV-2 vaccines were available in Germany. Samples were frozen at − 80 °C until use. CVID patient sera were kept at 3–5 °C o/n before freezing to allow completion of diagnostic tests first. Integrity of the complement system in those sera was verified by measuring CH50 before freezing. Only serum samples that were above the mean of the normal range of the test were used.

Three additional normal single donor complement-preserved serum samples (#ICERS10ML), and pooled cord blood serum samples (#IRHUCDS1ML) were obtained from Innovative Research, Inc, Novi, MI. Pooled serum was generated by mixing serum of 3 single donors. All donors gave informed written consent. This study was approved by the Ethics Committee of the Hannover Medical School (Ref. No: 3395-2016) and was performed in accordance with the ethical standards laid out in the 1964 Declaration of Helsinki and its later amendments.

Whenever crystals were in contact with buffer, HBSS containing 1.26 mM Ca^2+^, 0.9 mM Mg^2+^, and 5.5 mM d-glucose (Thermo Fisher Scientific, #14025050) was used. It was saturated with sodium urate to prevent dissolution of the MSU crystals.

### Immunoglobulins and CRP

Polyclonal and monoclonal human immunoglobulins used in the reconstitution experiments were obtained from Athens Research & Technology, Inc., Athens, GA (poly. IgM #16-16-090713; myeloma IgM #16-16-090713-M; poly. IgA #16-16-090701; poly IgG #16-16-090707). Purity of the antibodies was verified by SDS-PAGE (Supplementary Fig. 2a). Purified human CRP was from Merck KGaA (#AG723).

### Depletion of CRP

Where indicated, CRP-depleted serum was prepared by incubating 100 µl serum with p-Aminophenyl Phosphoryl Choline (PC)-agarose (ca. 5 µl packed beads) (Thermo Fisher Scientific, #20307) under mild agitation at 4 °C over-night. PC-agarose was removed by centrifugation at 2000×*g* for 2 min at 4 °C.

### Crystals and zymosan

**MSU crystals**: Lot 1 (small; mean length: 9.2 ± 4.4 µm): 10 mM uric acid (Merck KGaA, #U0881) was dissolved by boiling in ultrapure water containing 10 mM NaOH. After cooling to 60 °C, the pH was adjusted to 7.7, and 500 mM NaCl was added. Crystals formed under mild agitation at RT. They were harvested and either stored as dry powder at 4 °C or taken up in Hanks’ balanced salt solution (HBSS) at 50 mg/ml. Lot 2 (large; mean length: 66.8 ± 27.6 µm): 20 mM uric acid was dissolved by boiling in ultrapure water containing 20 mM NaOH. After cooling to 60 °C, the pH was adjusted to 8, and the solution was sterile filtered. Solutions were maintained under mild agitation at RT for 48 h. Crystals were harvested on a sterile filter, washed with ethanol p.a., dried overnight at 60 °C, taken up in phosphate-buffered saline (PBS) at 50 mg/ml, and stored at 4 °C or − 20 °C. An additional preparation was obtained from Invivogen (#tlrl-msu; lot MSU-41-01).

**t-CPPD** and **m-CPPD** were prepared as previously described^56^ using sodium pyrophosphate instead of potassium pyrophosphate as the starting material. Lot 1 was prepared in the laboratory of KN, and lot 2 was prepared in the laboratory of Christèle Combes, CIRIMAT, Université de Toulouse, Toulouse INP—ENSIACET, 31030, Toulouse, France.

**Silica** (Silicon dioxide) particles (− 325 mesh) were purchased from Alfa Aesar (#88316). Calcium carbonate crystals were prepared as described^26^.

**Zymosan** was purchased from Merck KGaA (#Z4250), washed 3× in 100% ethanol, stored in 100% ethanol at − 20 °C. Before use, zymosan was washed 3–4 times with HBSS.

### Analyses of protein binding to crystals

#### Flow cytometry

Crystals (100 µg) were incubated in 50 µl serum or HBSS + 10% BSA for 30 min at 37 °C with mild agitation. Crystals were harvested by centrifugation at 1000×*g* for 2 min and washed with HBSS. IgM and IgG were detected using PE anti-human IgM antibody (#314508, BioLegend) and PE anti-human IgG Fc antibody (#410708, BioLegend) (both diluted 1:25 in HBSS + 5% BSA), respectively. Particle fluorescence was analyzed using a FACS Canto II (BD Biosciences) and BD FACSDiva software version 6.1.3 (www.bdbiosciences.com). Flowing Software version 2.5.1 (Perttu Terho, University of Turku, Finland, https://bioscience.fi/services/cell-imaging/flowing-software/) was used for analysis of data obtained.

Fluorescence microscopy was performed as described before^26^ and images were acquired using an Olympus IX81 inverted microscope and CellR software (version 3.2; www.olympus-lifescience.com/en/software/). Brightness was adjusted, pseudo-color was inserted in the grayscale image, and scale bar was added using ImageJ (version 1.53e; https://imagej.nih.gov/ij/).

#### Western blot analysis

1 mg crystals were incubated in 50 µl serum for 30 min at 37 °C and harvested by centrifugation at 1000×*g* for 2 min (for LC–MS analysis 9 mg crystals were incubated with 100 µl serum).

Supernatants were directly diluted in reducing SDS-PAGE buffer (SDS, glycerol, DTT, Tris pH6.8), and crystals were washed 5 × in HBSS saturated with sodium urate and transferred to a fresh 1.5-ml tube before elution of the bound proteins using 40 µl of 2 × reducing SDS-PAGE buffer at 70 °C for 10 min.

Proteins were separated on precast SDS-PAGE gels (SERVA Electrophoresis GmbH, #43289) and transferred to nitrocellulose membranes (GE Healthcare, #10600003). The membranes were blocked in TBST + 5% BSA and incubated with primary antibody in TBST + 5% BSA o/n at 4 °C. After incubation with HRP-coupled secondary antibodies (Cell Signaling Technology, #7074, #7076 S), the blots were subjected to ECL reaction. Images were acquired using a ChemoStar imager (INTAS Science Imaging Instruments GmbH), and quantification of Western blot signals was performed with ImageJ software (version 1.53e).

The following primary antibodies were used at the indicated dilution: anti-C1S (#14554-1-AP; 1:4000), anti-C3/C3b/C3c (#21337-1-AP; 1:1000), anti-C5 (66634-1-Ig; 1:50,000), anti-IgA (11449-1-AP; 1:2000), anti-IgM (66484-1-Ig; 1:20,000) (all from Proteintech), anti-ApoB (#sc-393636; 1:200; SantaCruz Biotechnology), and anti-IgG (H + L) (peroxidase-coupled; #109-035-003; Jackson ImmunoResearch, 1:10,000).

For coomassie staining, gels were incubated in PageBlue (Thermo Fisher Scientific, #11852174).

### Quantification of C3a, C4a, C5a, and sC5b-9

1.5-ml polypropylene tubes were blocked with 30% heat-inactivated FBS at 4 °C o/n. Crystals (1 mg) were incubated in 50 µl of serum in these blocked tubes for 30 min at 37 °C with mild agitation. The supernatant was harvested and C3a, C4a, and C5a concentration was determined using the BD CBA human anaphylatoxin kit (BD Biosciences, 561418, sample dilution 1:15,000) and sC5b-9 concentration was determined using human TCC ELISA (Hycultec GmbH, HK328-02, sample dilution 1:750) according to the manufacturer’s recommendations.

### Statistical analysis

Unless otherwise stated in the figure legends, paired or unpaired Student’s two-tailed t-test was performed to compare the means of two groups. All analyses were performed using GraphPad Prism version 9 (GraphPad Software; www.graphpad.com/scientific-software/prism/). A p value of < 0.05 was considered statistically significant. Data generated using a flow cytometer represents the median fluorescent intensity of 5–10 k particles measured.

## Supplementary Information

Supplementary Information 1.

Supplementary Information 2.

## Data Availability

The data that supports the findings of this study are available in the Supplementary Information of this article or can be provided from the corresponding author upon reasonable request.

## Abbreviations

CPPD: Calcium pyrophosphate dihydrate
MSU: Monosodium urate
Ig: Immunoglobulin
sC5b-9: Soluble terminal complement complex
LC–MS: Liquid chromatography-coupled mass spectrometry
NHS: Normal human serum
FBS: Fetal bovine serum
t: Triclinic
m: Monoclinic
