# Supplementary material for: Natural antibodies and CRP drive anaphylatoxin production by urate crystals
Source: Sci Rep. 2022 Mar 16;12:4483. doi: 10.1038/s41598-022-08311-z (PMC8924570; doi:10.1038/s41598-022-08311-z)
Supplement: Supplementary file 2 — Supplementary Information 2. [file 41598_2022_8311_MOESM2_ESM.pdf]

Supplementary Information

Natural antibodies and CRP drive anaphylatoxin production by urate crystals

Anne Kathrin Wessig[1], Leonie Hoffmeister[1], Annika Klingberg[1], Anika Alberts[1], Andreas Pich[2], Korbinian Brand[1], Torsten Witte[3], Konstantin Neumann[1]*

[1]Institute of Clinical Chemistry, Hannover Medical School, 30625 Hannover, Germany
[2]Research Core Unit Proteomics & Institute of Toxicology, Hannover Medical School, 30625 Hannover, Germany
[3]Department of Immunology and Rheumatology, Hannover Medical School, 30625 Hannover, Germany

*Correspondence: E-mail: Neumann.Konstantin@mh-hannover.de; Telephone: +49-511-5325284; Fax: +49-511-5328614

Supplementary Figures

Supplementary Figure 1. Raw data of Fig. 1b
Supplementary Figure 2. Supplements to Fig. 1d and f
Supplementary Figure 3. Western blot of crystal-bound proteins from Fig. 3
Supplementary Figure 4a. Western blots used to quantify C1s in Fig. 5b and 5c
Supplementary Figure 4b. Western blots from Supplementary Fig. 4a without enhanced contrast or cropping
Supplementary Figure 5. Uncropped Western blots from Fig. 5a
Supplementary Figure 6. MSU crystal-induced activation of C1s by addition of polyclonal IgM

Supplementary Dataset

A dataset is attached as a separate EXCEL file:
Supplementary Dataset. LC-MS data of MSU crystal and zymosan-binding proteins

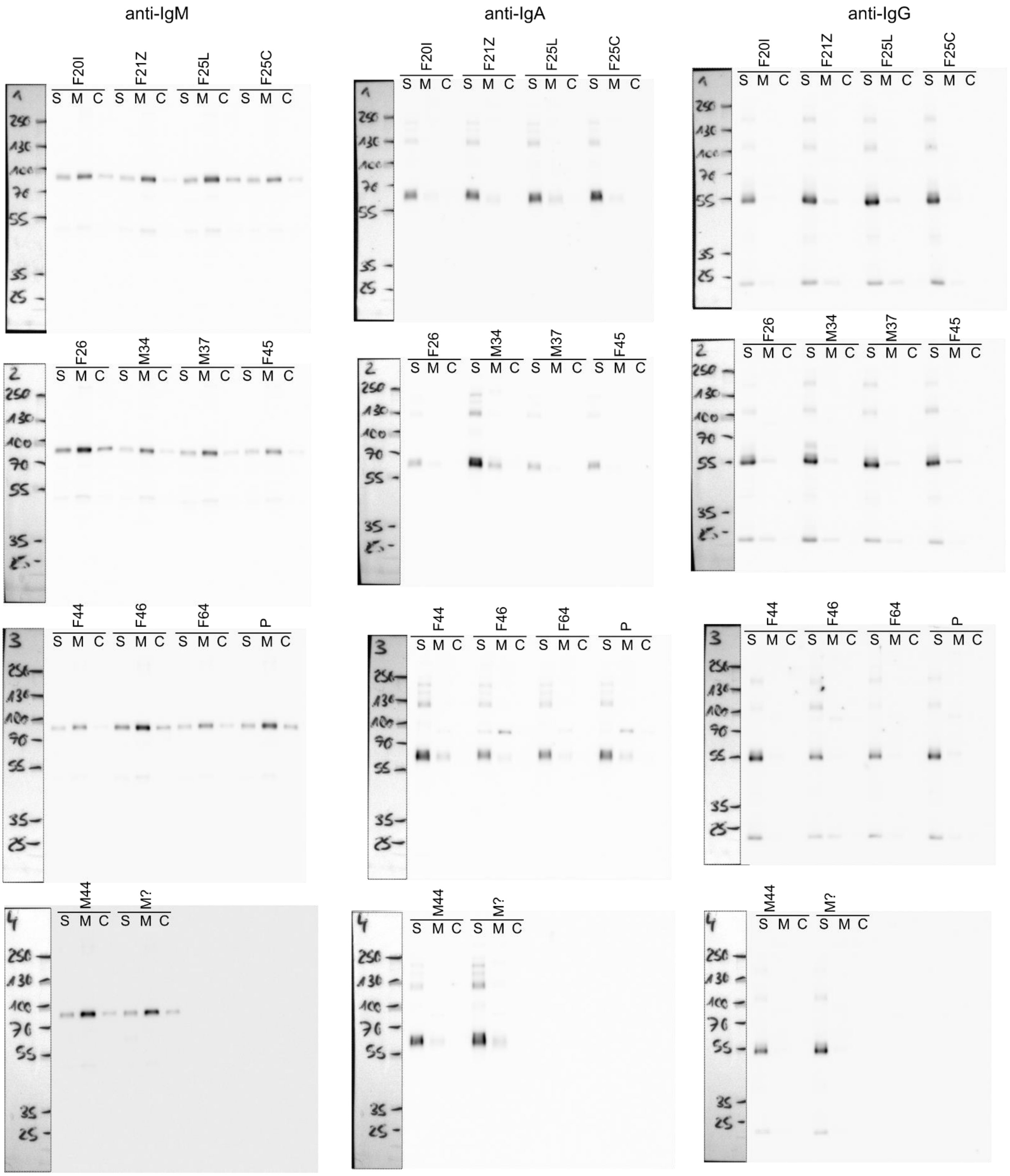

Supplementary Figure 1. Raw data of Fig. 1b

50 µl of 14 anonymized individual healthy donor serum samples were incubated with 2 mg MSU (M) or t-CPPD crystals (C), extensively washed, and bound proteins were eluted in 50 µl reducing SDS-PAGE buffer. 5 µl of the eluted proteins were applied to SDS-PAGE. In parallel, the same amount of a 1:20 dilution of the input serum (S) was applied. Gels were subjected to Western blot analysis using anti-IgM (left panel), anti-IgA (middle panel), or anti-IgG (right panel). Pictures were taken with a 16-bit CCD camera. The image of the prestained protein marker (PageRuler Plus) was pasted at the original position (indicated by dotted line).

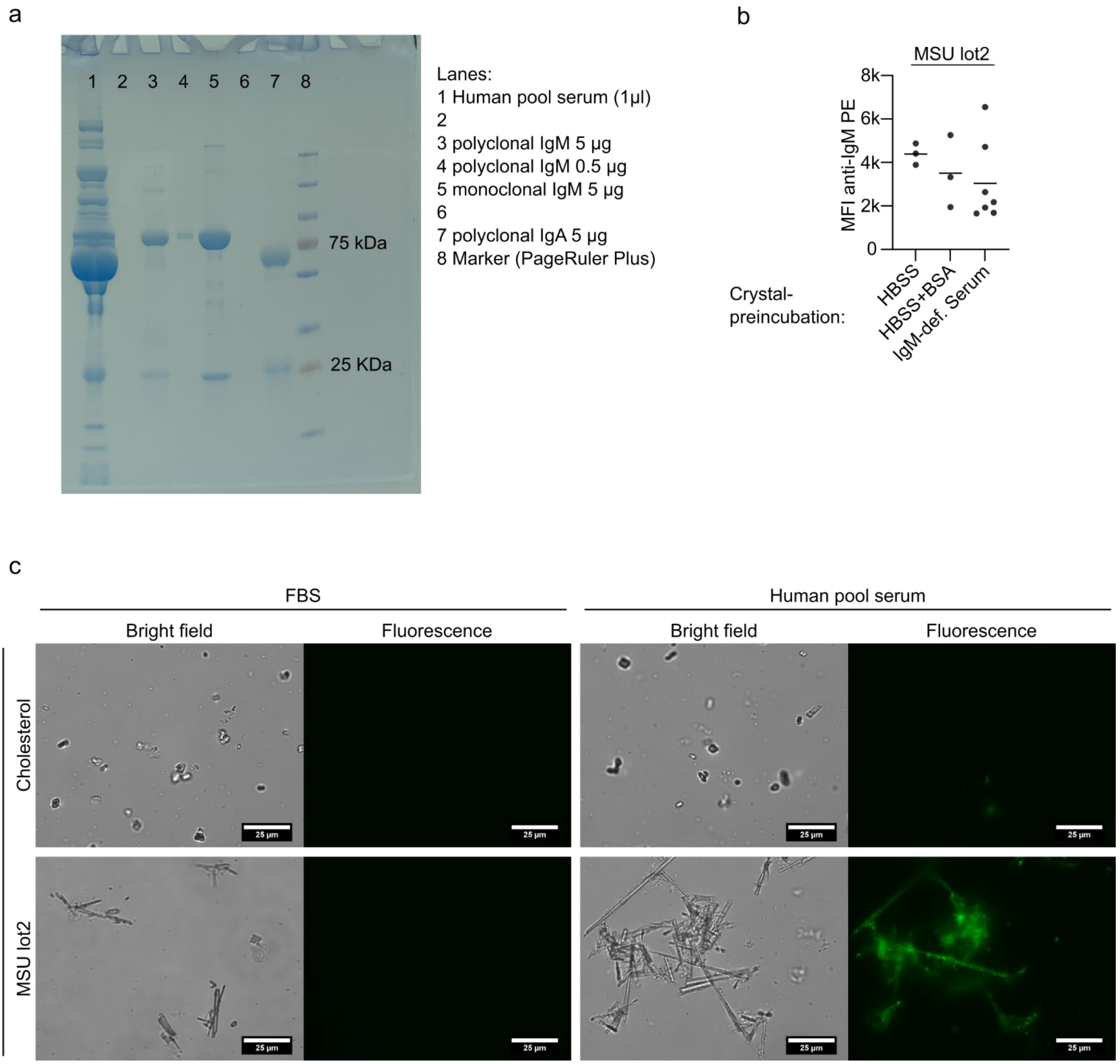

Supplementary Figure 2. Supplements to Fig. 1d and f

(**a**) Coomassie stained SDS-PAGE gel of purified immunoglobulins (polyclonal IgM and IgA, monoclonal IgM) used in Fig. 1d.

(**b**) Binding of IgM to opsonized crystals. MSU crystals (lot 2) were preincubated in IgM-deficient human serum (30 min, 37°C), then incubated in FBS (negative control) or human pool serum. Bound IgM was detected as in Fig. 1c and the resulting MFI was normalized by subtracting the MFI of the FBS-incubated samples.

(**c**) Indicated crystals were incubated with FBS (left panel) or human pool serum and bound human IgM was detected using AF488-coupled anti-human IgM.

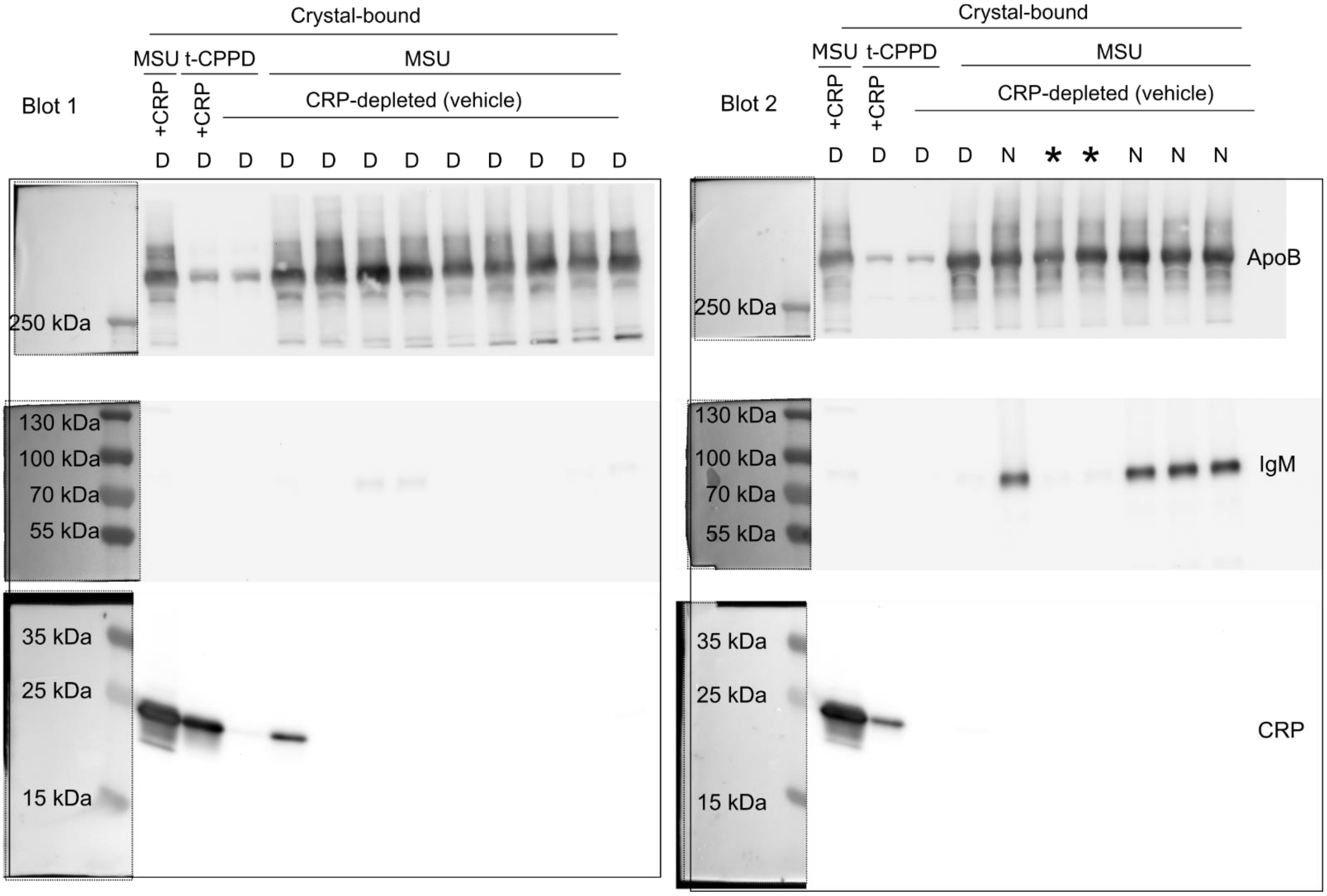

Supplementary Figure 3. Western blot of crystal-bound proteins from Fig. 3

For all serum samples described in Fig. 3, which were incubated with MSU crystals without addition of CRP (vehicle), crystal-bound proteins were eluted and applied to Western blot analysis. As controls, proteins eluted from MSU and t-CPPD crystals from CRP supplemented (30 µg/ml) serum samples are loaded on lane 1 and 2, respectively. Both blots were cut and developed with anti-ApoB, anti-IgM, and anti-CRP. The normal huma sera are marked with an N, the IgM/IgA-deficient sera are marked with a D, and the IgM/IgA-deficient sera that still activated early complement factors are marked by an asterix (*). Residual CRP was detected in one IgM/IgA-deficient serum (blot 1, lane 4). This CRP signal was much lower than in CRP-reconstituted sera (blot 1 and 2, lane 1). This serum showed the same response as the other IgM/IgA-deficient sera in complement activation by MSU crystals (Fig. 3).

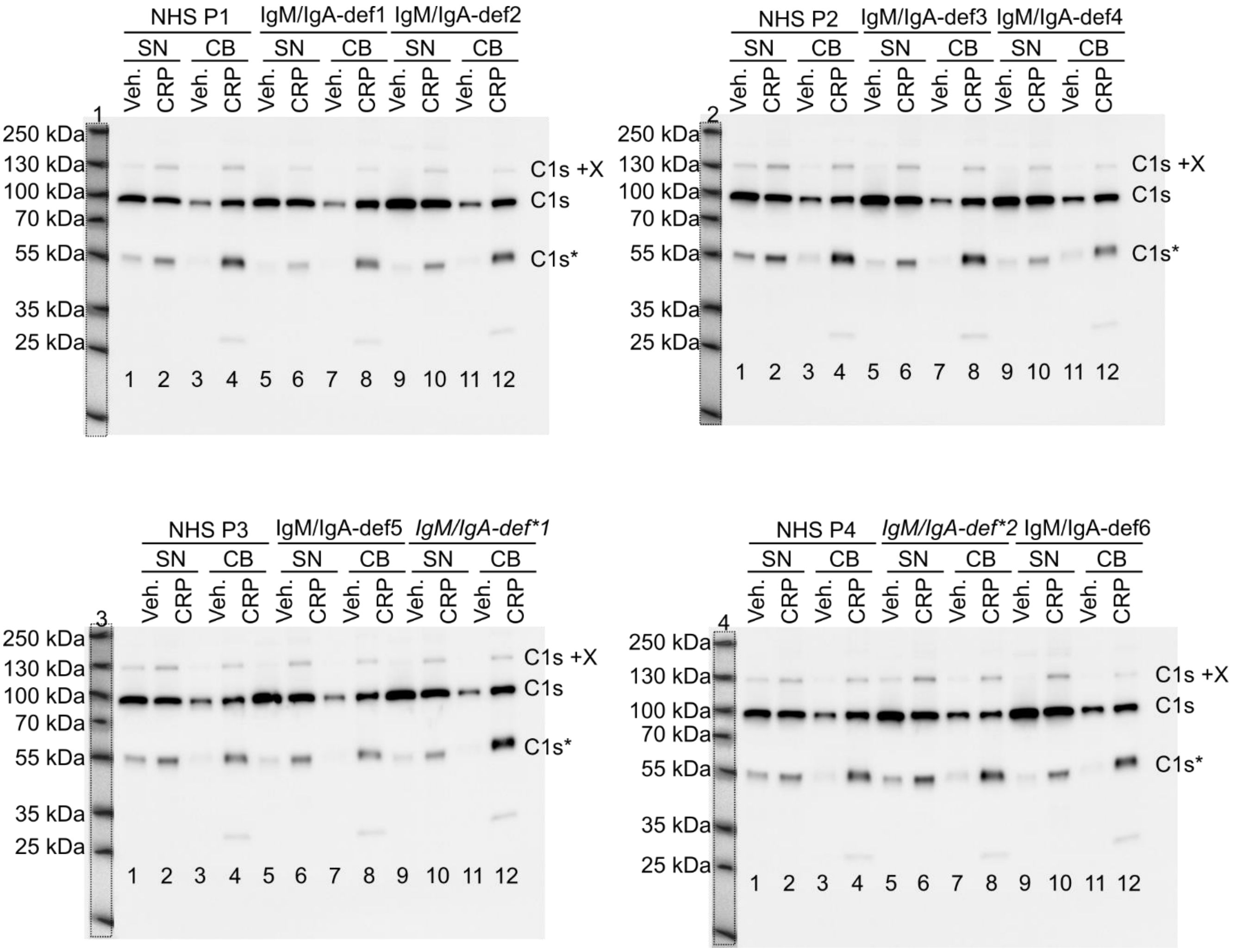

Supplementary Figure 4a. Western blots used to quantify C1s in Fig. 5b and 5c

Supernatants (SN) and crystal-bound (CB) proteins from 4 normal human pool serum samples (NHS P1 – P4) and 6 IgM/IgA-deficient sera were applied to Western blot analysis using C1s antibody. Additionally present on blot 3 and 4 are the two IgM/IgA-def.* sera (1&2) that showed some anaphylatoxin production in the absence of CRP (Fig. 3). These were not included in the quantification in Fig. 5b and 5c. Pictures were taken with a 16-bit CCD camera and the contrast was adjusted to enhance visibility of the bands of cleaved complement factors. The image of the prestained protein marker (PageRuler Plus) was pasted at the original position (indicated by dotted lines).

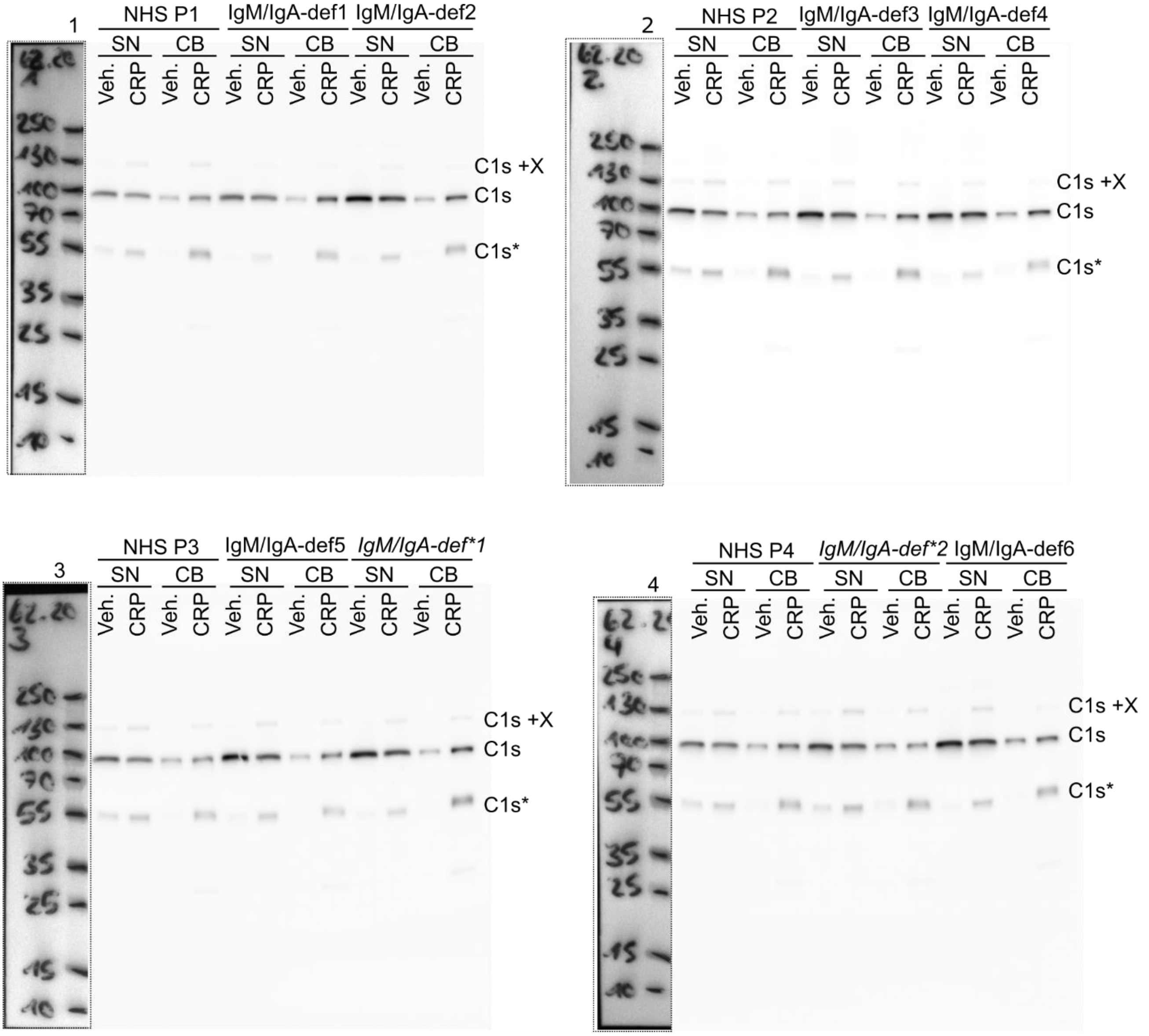

Supplementary Figure 4b. Western blots from Supplementary Fig. 4a without overexposure or cropping

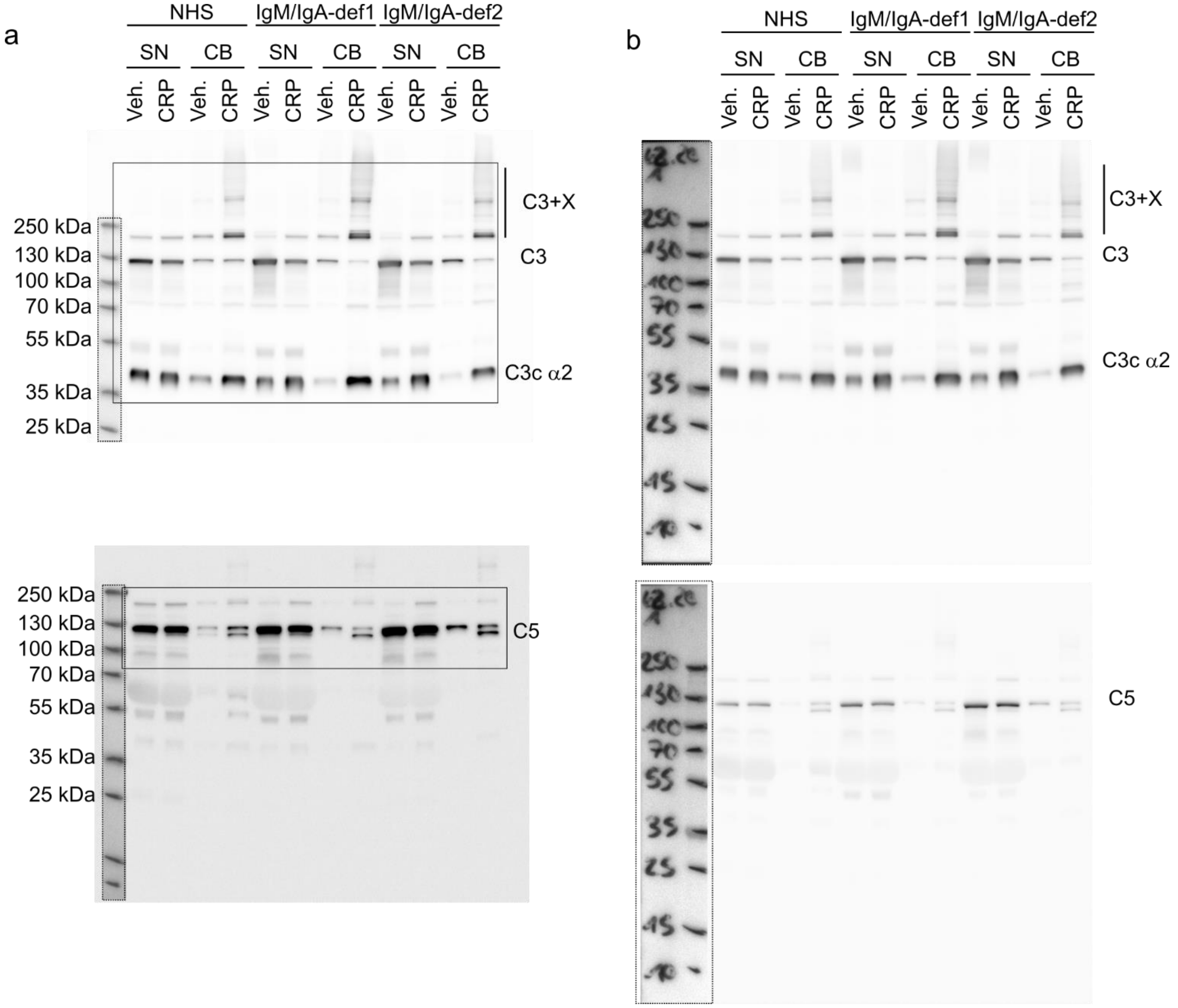

Supplementary Figure 5. Uncropped Western blots from Fig. 5a

(**a**) Uncropped Western blots of Fig. 5a are shown. Anti-C1s Western blots are already shown in Supplementary Fig. 4a and 4b. Pictures were taken with a 16-bit CCD camera and the contrast was adjusted to enhance visibility of the bands of cleaved complement factors. The image of the prestained protein marker (PageRuler Plus) was pasted at the original position (indicated by dotted lines). Borders used for cropping are indicated by solid lines. (**b**) Same Western blots as in (a) without enhanced contrast.

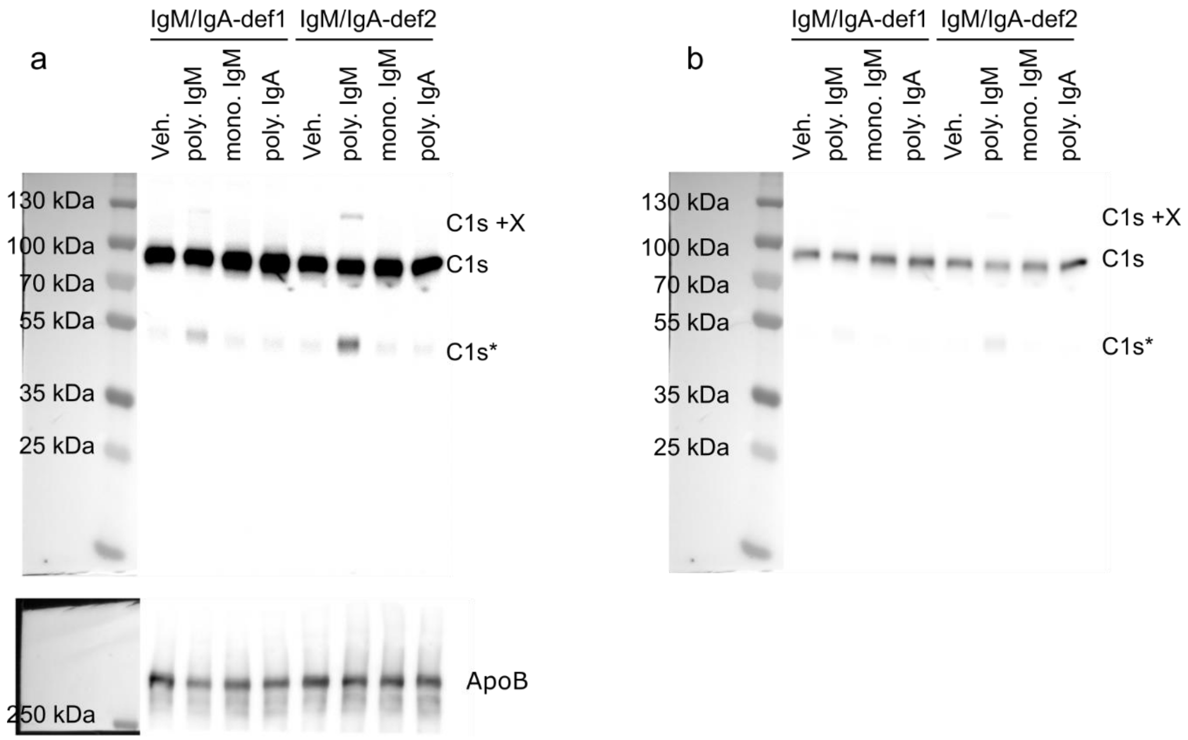

Supplementary Figure 6. MSU crystal-induced activation of C1s by addition of polyclonal IgM

(**a**) Two IgM/IgA-deficient serum samples were reconstituted with the indicated immunoglobulins (0.4 mg/ml) and incubated with MSU crystals for 30 min at 37°C. Supernatants were applied to Western blot analysis using anti-C1s and anti-ApoB. (**b**) Same blot for C1s as in (a) without enhanced contrast.
